# Attitudes toward Genetic Testing for Hypertension among African American Women and Girls

**DOI:** 10.1155/2013/341374

**Published:** 2013-11-02

**Authors:** Jacquelyn Y. Taylor, Bronwen Peternell, Jennifer A. Smith

**Affiliations:** ^1^Yale University, School of Nursing, P.O. Box 27399, West Haven, CT 06516-7399, USA; ^2^University of Michigan School of Public Health, Ann Arbor, MI, USA

## Abstract

*Introduction*. Although African American (AA) women have the highest prevalence of hypertension and many genetic studies have been conducted to examine this disparity, no published studies have investigated their attitudes toward genetic testing for hypertension. The purpose of the present study was to use the health belief model as a guide to examine attitudes toward perceived barriers and benefits of genetic testing held by AA multigenerational triads and to determine whether they differed by generation, age, education, or income level. *Methods*. A descriptive correlational research design were used with 183 African American women and girls from Detroit. Correlations between triad membership, age, income, and education level were examined for association with attitudes toward genetic testing. *Results*. Increasing age and education were associated with significant differences in attitudes regarding benefits (*F*[2, 160] = 5.19, *P* = 0.007, *d* = 0.06) and awareness (*F*[2, 160] = 6.49, *P* = 0.002, *d* = 0.08). No statistically significant differences existed on the three subscales when compared by income levels or triad membership. *Conclusions*. This highlights the need for increased outreach to younger generations regarding benefits of genetic services. Further research is necessary to determine whether rural and male populations have similar beliefs.

## 1. Introduction

Cardiovascular disease (CVD) carries the highest mortality rate for women in the United States. The American Heart Association (AHA) reports that more than 47% of African American women, of age 20 years and older, have been diagnosed with CVD as of 2008 [1]. Additionally, African American women also have the highest reported death rates from CVD [1]. Hypertension is a significant risk factor for the development of CVD [2]. Among African American women, 45.7% have hypertension marking them as possessing the highest incidence and prevalence rates amid all ethnic and racial groups in the United States [1]. In 2008, deaths related to hypertension among African American women totaled 7,002 with African American women's death rate being two and a half times more than that of Caucasian women [1, 3].

Research has shown that genetic factors contribute significantly to the susceptibility of developing hypertension [4–7]. A study of Caucasian and African American children revealed the T235 allele on the angiotensinogen gene to be more common in African American children compared to Caucasian. This is meaningful such that the T235 allele is positively correlated with increased serum angiotensinogen levels and hypertension in African American boys and girls, when compared to Caucasian children (*P* < 0.01) [8]. Other groups addressing African Americans have found that single-nucleotide polymorphisms (SNPs) on *SLC4A5*, a sodium bicarbonate transporter gene found on chromosome 2, were also significantly associated with hypertension [9–14]. Conversely, the presence of certain SNPs (such as SLC4A5 rs8179526) may be protective against the development of hypertension among African American women even when dietary sodium is elevated [13].

## 2. Genetic Testing

Genetic testing is a useful screening tool that can help identify people at high risk for developing disease and has the potential to enhance health and wellbeing [15]. However, research shows that few African Americans seek genetic services [16–19]. Studies have shown that fewer African American women seek genetic testing services compared to Caucasian women [20–22]. Underutilization of genetic services (including counseling and testing) reduces this group's ability to benefit from genetic testing, including early detection and intervention to prevent illness. To reduce disparities in the use of genetic services for heritable and preventable diseases, such as hypertension, efforts must be made to identify perceived barriers and attitudes towards genetic testing to ensure equal distribution of resources. By elucidating these barriers and attitudes among African American women and girls, the scientific and healthcare community can garner greater understanding to improve assessment and treatment of hypertension in this at-risk population. Currently, genetic testing for essential hypertension is not routinely conducted in the clinical setting. However, with-direct-to-consumer genetic testing for many disorders currently available, there is a growing potential for genetic testing to be used by the consumers to better inform their healthcare decision making for hypertension and many other diseases. The present study assesses the attitudes toward genetic testing for hypertension at a time when it is moving from a research question to more of a clinically relevant measure of health.

## 3. Conceptual Model

**Figure 1:**
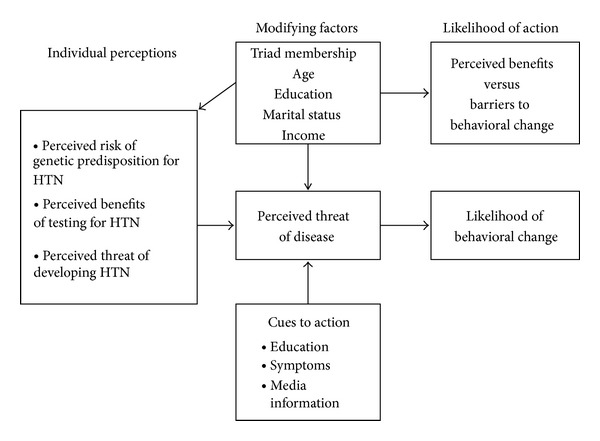
Health belief model.

To understand the factors that contribute to African American women and girls' decision to participate in genetic testing, the present study used aspects of the Health Belief Model (HBM) to guide its design. The HBM was originally developed to explain why individuals failed to participate in programs to detect and prevent disease (Figure 1) [23]. Researchers use the HBM to understand an individual's decision to take preventative measures, such as genetic testing, for personal health promotion [24]. The HBM considers an individual's perceived susceptibility to disease development as well as disease severity, personal demographic variables (gender, age, socioeconomic status, education, and knowledge), cues to action, and benefits and barriers regarding illness prevention [25]. Mobilization to act and take preventative measures is based on a cost-benefit analysis of all the components. 

The HBM includes four primary aspects that explain the infrequent acceptance of preventive practices and preillness screening tests: perceived susceptibility, perceived severity, perceived benefits, and perceived barriers [26]. Of the four components, Strecher and Rosenstock (1997) believe that perceived benefits and barriers are stronger predictors of the behavior change when the perceived threat is high [27]. Hypertension is a well-established risk factor for developing cardiovascular disease, a disease with high morbidity and mortality. Thus, the present study focused primarily on examining participants' perceived benefits, awareness, and outcomes of genetic testing, while considering certain demographic variables. 

The rationale for this approach was based on several components of the HBM [27]. First, a woman highly susceptible to hypertension may not undergo preventative actions, such as genetic testing, if the actions are not perceived to be efficacious. Likewise, if an action such as taking an antihypertensive medication is not believed to have positive consequences (e.g., decreasing risk for cardiovascular accident, heart attack, stroke, kidney disease, etc.), an action to take on other related lifestyle preventative measure (e.g., weight loss measures, increased physical activity, and dietary changes) may also be impacted. Finally, select demographic variables may have an indirect effect on health behaviors, thereby influencing personal perceptions of whether or not to implement preventative action. When personal demographics and perceived barriers are combined, they may affect a woman's decision.

Investigators have sought to understand reasons why African Americans participate less frequently in genetic testing than other ethnic groups by using the HBM, focusing primarily on two aspects—personal demographics and perceived barriers and benefits [24, 28]. Currently, no study exists which takes into account demographics such as age and education to assess perceived barriers and benefits associated with genetic testing for chronic diseases such as hypertension. The present study is also the first to address three generations of African American women and girls' attitudes toward benefits and barriers of genetic testing specific to hypertension. While research has been conducted addressing cancer and congenital issues with perceived barriers and benefits to genetic testing, the present study fills a gap in the literature by addressing a common complex disease such as hypertension in an at-risk population. 

Through phone interviews with over 800 African American and Caucasian participants (with known or unknown risk genetic risk for disease), Furr (2002) found that while age, gender, income, and educational achievement of African Americans held no influence on attitudes toward genetic testing in general, African Americans had increased negative perceptions regarding genetic testing when compared to European Americans [29]. Although studies have assessed attitudes/barriers to genetic testing for diseases such as ovarian, breast and colon cancer, the present study is the first study to examine participants' perceived benefits, awareness, and outcomes related to genetic testing for a chronic disease such as hypertension. Conversely in a later study, Forman and Hall (2009) found multiple socioeconomic barriers to genetic testing in women with breast and ovarian cancer [16]. Significant barriers included potential time constraints, limited access to knowledgeable providers, geographic barriers, limited awareness, language/cultural barriers, high cost, and ineligibility for Medicare/Medicaid. In yet another study, Sussner et al. (2009) found that foreign-born women of African descent reported more anticipation of negative emotional reactions about genetic testing for BRCA1/2 compared with US-born African American women [20]. Acculturation was suggested as being associated with perceived barriers and concerns regarding genetic testing. Acculturation is defined as the extent to which a majority culture is adopted by a minority culture thereby exchanging cultural elements, measured by length of time living in the US [20]. As these studies suggest, perceived barriers and benefits are significant in attitudes towards genetic testing. 

Common themes among African Americans in regards to genetic testing have been elucidated in recent research with lack of knowledge and/or negative attitudes towards genetic testing standing out as a predominant theme [20, 22, 29–31]. Thompson et al. (2002) assessed beliefs and attitudes towards breast cancer genetics research and found that knowledge of breast cancer genetics plays a major role in women's decision to undergo genetic testing [32]. This study was unique as authors viewed genetic testing as a process that began with counseling and ended with testing [32]. Authors recognized that refusal to participate in genetic testing could happen at any step (either before or after counseling) and that attitudes toward genetic testing may change between points of refusal.

In another cancer study, specifically focusing on hereditary colorectal carcinoma susceptibility, Kinney et al. (2001) used focus group interviews to obtain insight into beliefs, attitudes, and informational needs regarding genetic testing for patients with colorectal carcinoma or those with first-degree relatives diagnosed with this disease [33]. Though African Americans were undersampled, results revealed a general lack of knowledge regarding cancer genetics, genetic testing, concern over confidentiality issues, concern for other family members (specifically children), and the need to have a primary care provider be cognizant of these issues. In another focus group study, Murphy and Thompson (2009) studied predominantly African American participants with a history of anxiety or depression to assess attitudes, beliefs, and knowledge regarding genetic testing for psychiatric disorders [18]. Although a majority of participants lacked true understanding of genetic testing, participants had strong opinions concerning genetic use. Consensus was reached that genetic testing was beneficial, yet feared the process of testing could be harmful and painful.

Current literature supports conflicting attitudes and beliefs regarding genetic testing among African American women [34]. A review by Rew et al. (2009) noted few studies that focus directly on genetic testing in adolescents, remarking that of the limited available literature, educated Caucasian adolescents girls are oversampled [19]. Authors further argue that while ethical issues of genetic testing have been studied, empirical results to explain attitudes, beliefs, and knowledge of people who are participating in genetic testing are lacking especially in multigenerational families. As evidenced by the above-mentioned studies, methodologies used to assess psychosocial barriers to genetic testing vary. The present study uses one-on-one in-person interviews to obtain quantitative information.

While many studies have explored the psychosocial barriers to genetic testing for heritable diseases such as breast cancer, none have examined attitudes towards genetic testing for hypertension. The present study used several components of the Health Belief Model to better understand factors that influence African American women and girls when making a decision to undergo genetic testing for hypertension. Using specific personal demographic variables and perceptions of benefits and barriers associated with the health belief model, the present study examined attitudes toward genetic testing for hypertension among African American multigenerational triads (daughter-mother-grandmother). The research questions were as follows: (a) what are three generations of African American women and girls' attitudes toward genetic testing for hypertension? and (b) do African American women and girls' attitudes towards genetic testing for hypertension differ by triad (generation), age, education, or income level?

## 4. Methods

### 4.1. Design

The study employed a descriptive correlational research design to address the research questions. Correlations between variables (triad membership, age, income, and education) were examined to assess the strength and direction of the association with attitudes toward genetic testing (benefits, awareness, and outcomes). 

### 4.2. Sample and Setting

Participants in the present study included 183 African American women and girls from the Detroit Metropolitan area, originally involved in a parent study titled, “Hypertension and heredity: hypertension genetic polymorphisms in three generations of African American women” [35]. 

### 4.3. Human Subjects

Recruitment and the process of consent began after approval by University of Michigan and Wayne State University, Institutional Review Boards (IRB). Three generations of maternally, blood-related women and girls were recruited to examine genetic markers known to increase susceptibility to hypertension. Details of the research methods for the parent study are described elsewhere [35–38]. To meet the inclusion criteria for the parent study, participants were required to self-identify as African American and have a living family of at least three generations to constitute the triad of daughter-mother-grandmother. All participants who were recruited resided in urban and suburban neighborhoods in a large midwestern urban area. For those with a diagnosis of hypertension, their blood pressure had to average 140/90 or higher (stage 1 or 2 hypertension) without medication. Participants in the study also included: women diagnosed with diabetes, those who were on antihypertensive medication, or women who were normotensive and girls (offspring) not diagnosed with hypertension. Exclusion criteria consisted of having comorbidities of substance abuse, mental illness, end-stage cancer, end-stage renal disease, or other terminal illness. The researchers in the parent study excluded children under 12 years of age from completing the AGT survey because of the nature of the questions asked in the survey. 

### 4.4. Instruments

#### 4.4.1. Demographic Survey

Participants completed a research-developed questionnaire that collected information on family triad relationship (daughter, mother, grandmother), age, educational level, marital status, household income, and sources of income.

#### 4.4.2. Perceived Benefits and Barriers of Genetic Testing

The attitudes toward genetic testing (AGT) was developed by the PI of the present study. Ten items were used to measure participants' attitudes toward genetic testing for hypertension. The items were rated using a 4-point Likert-type scale ranging from 1 for strongly agree to 4 for strongly disagree. A principal components factor analysis with a varimax rotation was used to determine if subscales would emerge on the AGT. To be retained on a subscale, the factor loadings had to be greater than 0.40 and not load on more than one subscale. Three subscales (benefits, awareness, and outcomes) were emerged from the factor analysis, explaining 58.91% of the variance in AGT (see Table 1). The three subscales had eigenvalues greater than 1.00, indicating that they were each explaining statistically significant amounts of variance. Mean scores were obtained for each subscale and total scale for each participant by summing the responses and dividing by the number of items with valid responses. Lower scores were indicative of more positive attitudes toward genetic testing. The internal consistency of the AGT was tested using Cronbach's alpha. The obtained coefficient of 0.66 provided evidence that the instrument had adequate internal consistency. Face validity of the instrument was determined by having three experts on genetic testing review the instrument. They indicated that the instrument had good face validity.

### 4.5. Statistical Analysis

A one-way multivariate analysis of variance (MANOVA) and a one-way analysis of variance (ANOVA) were used to determine if there was any significant difference between triad members (daughters, mothers, grandmothers) on their attitudes toward genetic testing. 

Data collected from the included triads were analyzed using PASW Statistics, ver. 17.0 (SPSS, Inc., Chicago, IL, USA). The data analysis included descriptive statistics to summarize responses to age, number of children and grandchildren, marital status, level of education, household income, and sources of income. Measures of central tendency summarized demographic information. 

A one-way analysis of variance (ANOVA) was used to determine if any differences could be found among the triad members' (daughters, mothers, grandmothers) attitudes regarding genetic testing. The three subscales (benefits, awareness, and outcomes) from the AGT survey were used as dependent variables in a one-way multivariate analysis of variance (MANOVA) to determine if there were differences among the triads by income, educational level, and age. All decisions on the statistical significance of the findings were made using an alpha level of 0.05. Given the sample size (*N* = 183), power analysis showed that we had >80% power to detect effect sizes as low as 0.23 at a significance level of 0.05. The “*d*” is the dimension of the group means, or an estimate of the effect size, that represents the practical significance. The “*d*” ranges from 0 to 1, with numbers closer to 1 representing a stronger effect. Practical significance provides the reader with the importance of the findings to clinical practice based on effect size even when the results may not be statistically significant. Statistical significance has a greater dependence on sample size than effect size and is the standard method of determining important differences between variables in research studies. When conducting research in nursing science, it is important to represent both of these statistics in the findings. 

## 5. Results

### 5.1. Age

A total of 183 participants were included in the study. Of this number, 45 (24.6%) were grandmothers, 69 (37.7%) were mothers, and 69 (37.7%) were daughters. The participants ranged in age from 12 years to 93 years. The mean age of the grandmothers was 65.64 years (SD = 12.30). The mothers had a mean age of 46.39 years (SD = 15.17). The average of the granddaughters' age was 21.95 years (SD = 16.13). 

### 5.2. Educational Level

The educational levels of the grandmothers were generally high school (*n* = 11, 24.4%) or some college (*n* = 12, 26.7%). In contrast, the largest group of mothers (*n* = 27, 39.1%) had completed some college, with 16 (23.3%) indicating they had attained bachelor degrees. The granddaughters' ages indicated that a substantial proportion had not yet completed high school (*n* = 28, 40.7%). One (2.2%) grandmother and 2 (2.9%) granddaughters had obtained doctorate degrees (see Table 2).

### 5.3. Marital Status

Grandmothers were more likely to be widowed (*n* = 15, 33.3%) or married (*n* = 11, 24.5%), while mothers tended to be either married (*n* = 23, 33.3%) or single (*n* = 20, 29.1%). Most of the granddaughters tended to be single (*n* = 54, 78.4%).

### 5.4. Income

The household incomes of the grandmothers ranged from less than $10,000 to over $80,000. The largest group of grandmothers had incomes between $10,000 and $40,000 (*n* = 23, 51.2%). The largest group of mothers had incomes between $40,000 and $60,000 (*n* = 14, 20.4%). The largest group of granddaughters who were working had income levels that ranged from $40,000 to $60,000 (*n* = 15, 21.8%). 

Participants were provided with a list of possible sources of income and were asked to indicate all that applied. As a result, the number of responses was greater than the number of participants. The sources of income for grandmothers were mostly from Social Security (*n* = 24, 53.3%) or retirement/pension (*n* = 20, 44.4%). Sixteen (35.6%) grandmothers received income from working. The majority of the mothers were working (*n* = 47, 68.1%), with 14 (20.3%) indicating they were receiving Social Security. Twenty-nine (43.9%) of the granddaughters indicated they were receiving income from working.

### 5.5. ANOVA by Triad Membership

Results of the one-way analysis of variance comparing total scores on the AGT by triad membership were not statistically significant (*F* [2,160] = 1.27, *P* = 0.283). This finding provided support that attitudes toward genetic testing did not differ relative to the generation being asked. Grandmothers (M = 1.65, SD = 0.36), mothers (M = 1.54, SD = 0.34), and granddaughters (M = 1.61, SD = 0.39) had similar positive attitudes toward genetic testing. 

### 5.6. MANOVA by Age

The comparison of the three subscales (benefits, awareness, and outcomes) associated with attitudes toward genetic testing were used as dependent variables in a one-way MANOVA, with age of the participants used as the independent variable (see Table 3). The results of this analysis were statistically significant, *F* [6,316] = 3.90, *P* = 0.001, *d* = 0.07. When the three subscales were examined separately, benefits, *F* [2,160] = 5.19, *P* = 0.007, *d* = 0.06, and awareness, *F* [2,160] = 6.49, *P* = 0.002, *d* = 0.08, differed significantly. The participants who were between 22 and 50 years of age (M = 1.28, SD = 0.40) had significantly more positive attitudes regarding benefits of genetic testing than participants who were 21 years and younger (M = 1.62, SD = 0.54). The participants who were between 22 and 50 years of age (M = 1.16, SD = 0.35) and those who were over 50 years of age (M = 1.18, SD = 0.45) had significantly more positive attitudes about awareness of genetic testing than those who were 21 years and younger (M = 1.60, SD = 0.97) (see Table 4).

### 5.7. MANOVA by Educational Level

The results of the one-way MANOVA used to compare the three subscales measuring AGT by the educational level of the participants was statistically significant (see Table 5), *F* [15, 425.53] = 3.01, *P* = 0.001, *d* = 0.09. Statistically significant differences were obtained for benefits, *F* [5,156] = 3.66, *P* = 0.004, *d* = 0.11, and awareness, *F* [5,156] = 5.86, *P* < 0.001, *d* = 0.16. Participants who had a graduate degree (M = 1.15, SD = 0.24) had significantly more positive attitudes regarding the benefits of genetic testing than those who had completed high school or obtained a GED (M = 1.61, SD = 0.60). The participants who had not completed high school (M = 1.84, SD = 1.04) had significantly poorer attitudes regarding their awareness of genetic testing than participants with the other five educational levels. No statistically significant differences were found among the participants on the three subscales measuring AGT when compared by income levels or triad membership. 

## 6. Discussion

The present study found that urban African American women and girls across multiple generations were aware of the benefits and outcomes of genetic testing correlating with increased age and education level. These findings were similar to previous research by Murphy and Thompson (2009) who found African Americans to believe that genetic testing is beneficial but lacked understanding of the process itself [30]. In the present study, positive attitudes toward awareness and perceived benefits of genetic testing increased with age and level of education, possibly due to increased exposure through life experiences and/or education. These findings contradict Donovan and Tucker's research (2000) in which education levels were unrelated to the degree of knowledge regarding the genetics of heritable disease [34]. The Health Belief Model asserts that knowledge and understanding of perceived benefits must outweigh the risks if preventative action (such as genetic testing) is to be employed. Specific education to reach a less informed younger generation is indicated to expand knowledge in benefits of genetic testing. 

A unique component of the present study's research design was the recruitment of three generations of women in African American families. The African American family structure has been described as matriarchal one in which the eldest woman often makes health care decisions for the family [39]. Based on the results of the present study, the most likely family member to have the least amount of knowledge and, thus, decline the testing would be the daughter. However, because of the strong maternal hierarchy of the family, the younger generation typically conforms to the grandmother's wishes. Respect for the eldest female family member could be a contributing factor for African American women and girls to participate in genetic testing for hypertension. African Americans over 50 years of age need to be apprised of the guidelines and policy recommendations for ethical use of genetic testing on families and children [40, 41].

Although research has shown that while some African Americans have lower incomes, higher unemployment, and less access to health insurance and medical care compared to Caucasians, income level was not associated with attitudes towards genetic testing in the present study [42]. Forman and Hall (2009) suggest that cost, availability, lower socioeconomic status, and limited access to health care and preventive services are a significant barrier to genetic testing [16]. Focusing though solely on economic issues ignores the multifactorial nature of barriers to genetic services among African Americans such as age, education, and family structure which were significant in the present study [43]. 

## 7. Limitations

The women in the present study gave prior consent to being genetically tested and, therefore, their perceptions may be substantially biased towards positive acceptance. Additionally, the attitudes expressed in this paper are reflections by African American women and girls only and cannot be generalized to women of other ethnic groups or men. Findings may also not be generalizable to those who live in other geographic areas, as the study population was recruited from a large urban midwestern city. As the survey incorporated only ten items, the AGT questionnaire may not address the entire gamut of psychosocial barriers and perceptions held by participants. Because the overall Cronbach alpha for the AGT instrument was 0.66 and the commonly acceptable level is 0.70, we recognize this as a possible limitation in the study. However, after conducting factor analysis for the three subscales, we determined that each of the factor loadings was greater than 0.40 and did not load on more than one subscale. Three subscales (benefits, awareness, and outcomes) emerged from the factor analysis explaining 58.91% of the variance in AGT. The three subscales had eigenvalues greater than 1.00, indicating that they were each explaining statistically significant amounts of variance and were adequate. 

In addition, genetic testing is not commonly used clinically for essential hypertension and, therefore, based on the HBM, responses may be impacted by the fact that this testing is not commonly used in practice. However, based on this and future studies, this trend in lack of testing for chronic disease such as hypertension for the use in the health care setting for health care-related decision making could be changing. 

## 8. Conclusions

Diverse barriers to genetic risk assessment exist for African American women and girls. By elucidating perceived barriers to genetic testing by African American women and girls, health care providers can design gender-specific, culturally relevant services for outreach, genetic counseling and testing to promote early and appropriate intervention for an at-risk population. Genetic testing has the potential to reveal specific markers that may identify risk for, or protection against, the development of hypertension. By identifying such markers prior to the condition's onset, more meaningful genetic counseling can be delivered to family members. Likewise, a genetic test has the potential to provide information to an individual diagnosed with hypertension on how to best manage the condition. Greater participation by African American women and girls in genetic testing can provide a better foundation for knowledge regarding the etiology of hypertension in this population as well as its appropriate management. 

The present study highlights the complex nature of an individual's decision to pursue genetic testing. For these women, openness to undergo hypertension risk assessment may have been influenced by familial, educational, and age-related factors. Further investigation is needed in each of these subcategories to understand how they contribute to African American women and girls' perceptions towards genetic testing. This information could shape specific outreach to address corresponding gaps in knowledge and understanding of genetic testing. As the present study represented the beliefs of African American women and girls from an urban metropolitan area, additional research is indicated to clarify motivations for pursuing genetic testing in hypertension across other settings and groups, so that healthcare providers can best guide prevention and intervention efforts. 

## Figures and Tables

**Table 1:** Principal components factor analysis of “Attitudes toward genetic testing (AGT)”.

Item	Factor		
	Benefits	Awareness	Outcomes
(1) You are confident that the results of your genetic tests will be kept confidential.	0.58		
(2) Genetic testing is relevant to you and/or your child's health.	0.57		
(3) Genetic testing is beneficial in the prevention of the disease.	0.85		
(4) Genetic testing is beneficial in the treatment of the disease.	0.86		
(5) Genetic testing is beneficial in preventing the disease.	0.68		
(6) You would like to know if you and/or your child tests positive for a genetic disorder.		0.78	
(7) If you or your child tested positive for a genetic disorder, you would seek medical care immediately to minimize you or your child's chances of developing the disease.		0.89	
(8) If you or your child tested positive for a genetic disorder, would you wait until you and/or your child experienced signs and symptoms of the disease before obtaining medical care?			0.63
(9) If you or your child tested positive for a genetic disorder, you believe that you and/or your child would be treated differently by healthcare providers.			0.73
(10) If you test positive for a genetic disorder, it is likely that your child is at risk for testing positive for the same disease.			0.60
Percent of explained variation	26.86	18.47	13.58
Eigenvalues	2.69	1.85	1.36

**Table 2:** Personal characteristics by triad membership.

Personal characteristics	Triad membership						Total (*N* = 183)	
	Grandmother (*n* = 45)		Mother (*n* = 69)		Granddaughter (*n* = 69)			
	*N*	%	*N*	%	*N*	%	*N*	%
Age (years)								
≤18	0	0	0	0	35	50.7	35	19.1
19–24	0	0	1	1.5	12	17.3	13	7.1
25–34	0	0	17	24.7	5	7.2	22	12.0
35–44	1	2.2	19	27.5	8	11.6	28	15.3
45–54	10	22.2	15	21.7	7	10.1	32	17.5
55–64	9	20.0	8	11.6	2	2.9	19	10.4
65+	25	55.6	9	13.0	0	0	34	18.6
Educational level								
Less than high school	5	11.1	2	2.9	28	40.7	35	19.1
High school/GED	11	24.4	9	13.0	11	15.9	31	16.9
Some college	12	26.7	27	39.1	8	11.6	47	25.8
Associate degree	7	15.6	3	4.3	4	5.8	14	7.7
Bachelor degree	4	8.9	16	23.3	13	18.8	33	18.0
Master degree	4	8.9	12	17.4	2	2.9	18	9.8
Doctorate	1	2.2	0	0.0	2	2.9	3	1.6
Missing	1	2.2	0	0.0	1	1.4	2	1.1
Marital status								
Married	11	24.5	23	33.3	9	13.0	43	23.5
Single	4	8.9	20	29.1	54	78.4	78	42.6
Divorced	10	22.2	17	24.6	5	7.2	32	17.5
Separated	4	8.9	2	2.9	0	0.0	6	3.3
Widowed	15	33.3	5	7.2	0	0.0	20	10.9
Missing	1	2.2	2	2.9	1	1.4	4	2.2
Household income								
Less than 10 k	8	17.7	8	11.6	14	20.3	30	16.4
10 k to 20 k	9	20.0	7	10.1	6	8.7	22	12.0
20 k to 30 k	7	15.6	8	11.6	6	8.7	21	11.5
30 k to 40 k	7	15.6	11	15.9	7	10.1	25	13.7
40 k to 60 k	6	13.3	14	20.4	15	21.8	35	19.1
60 k to 80 k	3	6.7	10	14.5	9	13.0	22	12.0
80 k and higher	3	6.7	8	11.6	8	11.6	19	10.4
Missing	2	4.4	3	4.3	4	5.8	9	4.9
Sources of income*								
Wages from employment	16	35.6	47	68.1	29	43.9	92	51.1
Social Security	24	53.3	14	20.3	2	3.0	40	22.2
Retirement/pension	20	44.4	10	14.5	3	4.5	33	18.3
IRA/401 Ks	2	4.4	2	2.9	0	0.0	4	2.2
Welfare	1	2.2	4	5.8	4	6.1	9	5.0
Investments	3	6.7	4	5.8	0	0.0	7	3.9
Other sources of income	6	13.3	9	13.0	36	54.5	51	28.3

*Participants were encouraged to indicate more than one source of income if appropriate.

**Table 3:** One-way MANOVA—subscales measuring attitudes toward genetic testing by triad membership.

Triad	Subscales					
	Benefits		Awareness		Outcomes	
	M	SD	M	SD	M	SD
Grandmother	1.50	0.51	1.18	0.44	2.20	0.56
Mother	1.33	0.44	1.17	0.40	2.14	0.50
Granddaughter	1.38	0.46	1.35	0.71	2.16	0.62

MANOVA *F* ratio: *F* [6,316] = 1.55, *P* = 0.162, *d* = 0.03 (based on Wilk's lambda).

Between subjects: benefits: *F* [2,160] = 1.86, *P* = 0.159, *d* = 0.02.

Awareness *F* [2,160] = 1.96, *P* = 0.145, *d* = 0.02.

Outcomes *F* [2,160] = 0.19, *P* = 0.830, *d* =< 0.01.

Note: Lower scores indicate more positive perceptions of attitudes toward genetic testing.

Twenty granddaughters were less than 12 years of age and did not complete the attitudes toward genetic testing survey.

**Table 4:** One-way MANOVA—subscales measuring attitudes toward genetic testing by age of participants.

Age	Subscales					
	Benefits		Awareness		Outcomes	
	M	SD	M	SD	M	SD
21 years and younger	1.62_a_	0.54	1.60_a,b_	0.97	2.29	0.53
22 to 50	1.28_a_	0.40	1.16_a_	0.35	2.05	0.53
Over 50 years	1.45_a_	0.49	1.18_b_	0.45	2.25	0.57

MANOVA *F* ratio: *F* [6,316] = 3.90, *P* = 0.001, *d* = 0.07 (based on Wilk's lambda).

Between subjects: benefits: *F* [2,160] = 5.19, *P* = 0.007, *d* = 0.06.

Awareness *F* [2,160] = 6.49, *P* = 0.002, *d* = 0.08.

Outcomes *F* [2,160] = 3.01, *P* = 0.052, *d* = 0.04.

Note: Lower scores indicate more positive perceptions of attitudes toward genetic testing.

Means in a column sharing subscripts are significantly different.

Twenty granddaughters were less than 12 years of age and did not complete the attitudes toward genetic testing survey.

**Table 5:** One-way MANOVA—subscales measuring attitudes toward genetic testing by educational level of participants.

Educational level	Subscales					
	Benefits		Awareness		Outcomes	
	M	SD	M	SD	M	SD
Less than high school	1.60	0.53	1.84_a,b,c,d,e_	1.04	2.40	0.51
High school/GED	1.61_a_	0.60	1.21_a_	0.60	2.18	0.54
Some college	1.35	0.42	1.15_b_	0.31	2.11	0.49
Associate degree	1.40	0.48	1.18_c_	0.32	2.00	0.55
Bachelor degree	1.29	0.38	1.17_d_	0.32	2.10	0.61
Graduate degree	1.15_a_	0.24	1.07_e_	0.24	2.25	0.63

MANOVA *F* ratio: *F* [15,425.53] = 3.01, *P* = 0.001, *d* = 0.09 (based on Wilk's lambda).

Between subjects: benefits: *F* [5,156] = 3.66, *P* = 0.004, *d* = 0.11.

Awareness *F* [5,156] = 5.86, *P* < 0.001, *d* = 0.16.

Outcomes *F* [5,156] = 1.12, *P* = 0.353, *d* = 0.04.

Note: Lower scores indicate more positive perceptions of attitudes toward genetic testing.

Means in a column sharing subscripts are significantly different.

Twenty granddaughters were less than 12 years of age and did not complete the attitudes toward genetic testing survey.
